# Oral intake of *Lactobacillus plantarum* L‐14 extract alleviates TLR2‐ and AMPK‐mediated obesity‐associated disorders in high‐fat‐diet‐induced obese C57BL/6J mice

**DOI:** 10.1111/cpr.13039

**Published:** 2021-04-08

**Authors:** Jaehoon Lee, Sangkyu Park, Naeun Oh, Jaehyun Park, Mijin Kwon, Jeongmin Seo, Sangho Roh

**Affiliations:** ^1^ Biomedical Research Institute NeoRegen Biotech Co., Ltd. Seoul Korea; ^2^ Cellular Reprogramming and Embryo Biotechnology Laboratory Dental Research Institute Seoul National University School of Dentistry Seoul Korea

**Keywords:** AMPK signalling pathway, exopolysaccharide, *Lactobacillus plantarum*, metabolic disorders, obesity, Toll‐like receptor 2

## Abstract

**Objectives:**

Whether periodic oral intake of postbiotics positively affects weight regulation and prevents obesity‐associated diseases in vivo is unclear. This study evaluated the action mechanism of *Lactobacillus plantarum* L‐14 (KTCT13497BP) extract and the effects of its periodic oral intake in a high‐fat‐diet (HFD) mouse model.

**Materials and methods:**

Mouse pre‐adipocyte 3T3‐L1 cells and human bone marrow mesenchymal stem cells (hBM‐MSC) were treated with L‐14 extract every 2 days during adipogenic differentiation, and the mechanism underlying anti‐adipogenic effects was analysed at cellular and molecular levels. L‐14 extract was orally administrated to HFD‐feeding C57BL/6J mice every 2 days for 7 weeks. White adipose tissue was collected and weighed, and liver and blood serum were analysed. The anti‐adipogenic mechanism of exopolysaccharide (EPS) isolated from L‐14 extract was also analysed using Toll‐like receptor 2 (TLR2) inhibitor C29.

**Results:**

L‐14 extract inhibited 3T3‐L1 and hBM‐MSC differentiation into mature adipocytes by upregulating AMPK signalling pathway in the early stage of adipogenic differentiation. The weight of the HFD + L‐14 group (31.51 ± 1.96 g) was significantly different from that of the HFD group (35.14 ± 3.18 g). L‐14 extract also significantly decreased the serum triacylglycerol/high‐density lipoprotein cholesterol ratio (an insulin resistance marker) and steatohepatitis. In addition, EPS activated the AMPK signalling pathway by interacting with TLR2, consequently inhibiting adipogenesis.

**Conclusions:**

EPS from L‐14 extract inhibits adipogenesis via TLR2 and AMPK signalling pathways, and oral intake of L‐14 extract improves obesity and obesity‐associated diseases in vivo. Therefore, EPS can be used to prevent and treat obesity and metabolic disorders.

## Graphical abstract

EPS regulates lipid accumulation and glucose uptake via TLR2 and AMPK signalling pathway. When EPS binds to TLR2 in the cell membrane, MyD88, a downstream adaptor protein of TLR, initiating AMPK signalling cascades. The phosphorylated AMPKα inactivates SREBP‐1c, ACC, FAS and SCD1 by phosphorylation. In addition, the activated AMPKα inhibits the expression of core adipogenesis markers including PPARγ, C/EBPα and FABP4. The reduced PPARγ expression decreases the expression of adiponectin. These changes eventually reduce lipid accumulation in adipocytes. Also, EPS induces phosphorylation of AKT through a direct or indirect pathway and the activated AKT and AMPKα phosphorylates AS160. The activated AS160 can allow GLUT4 translocation to plasma membrane and subsequently enhance glucose uptake.

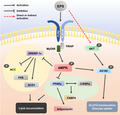

## 1 INTRODUCTION

According to World Health Organization, in 2016, ~13% of the world's adult population and >3.4 million children and adolescents were obese.^1^ Obesity is defined as a condition in which abnormal or excess fat accumulates and becomes a risk factor for numerous chronic diseases, such as hypertension, type 2 diabetes, cardiovascular diseases and mental disorders.^2^, ^3^, ^4^ Combined diseases due to obesity are strongly associated with increased mortality.^5^ Obesity also imposes an enormous economic burden on individuals and nations, in addition to being a significant public health concern.^6^ Meta‐analysis shows that in 2014, the annual medical expenditure of patients of obesity was $1239‐$2582, accounting for $149.4 billion at the national level.^7^ Therefore, the prevention or treatment of obesity is necessary from the public health and economic point of view.

Extra calories consumed grow into fat via an increase in the size and number of adipocytes.^8^ Adenosine monophosphate (AMP)‐activated protein kinase (AMPK) is involved in maintaining the cellular energy balance and homeostasis of lipids and cholesterol in the body. When AMP/adenosine triphosphate (ATP) and adenosine diphosphate (ADP)/ATP ratios increase, the body activates AMPKα to meet its cellular energy demands. Liver‐specific AMPK activation prevents triacylglycerol (TAG) accumulation in the liver.^9^ Leptin released from adipocytes produces a feeling of satiety and stimulates fatty acid oxidation by activating the AMPK signalling pathway in muscle.^10^ However, obesity leads to chronic inflammation in the body, and matrix metalloproteinase‐2 (MMP2), which is activated by nuclear factor kappa B (NF‐κB) in hypothalamic cells, induces leptin resistance by cleaving hypothalamic leptin receptors.^11^ This leptin resistance inhibits the AMPK signalling pathway, causing failure of systemic metabolism homeostasis and aggravating obesity and inflammation.^12^ In addition, functional failure of AMPK induces insulin resistance through mammalian target of rapamycin pathways,^13^ and liver‐specific AMPK activation decreases steatohepatitis,^9^ indicating that targeting the AMPK signalling pathway can be a potential method of treating or preventing obesity.

Although obesity is due to complex interactions of genetic, environmental and other factors, bacteria have been used to treat obesity since it was found that obesity is closely related to specific functional configurations of the gut microbiota.^14^ *Lactobacillus plantarum* (*L. plantarum*) is a versatile lactic acid bacterium with beneficial effects, such as decreasing irritable bowel syndrome symptoms,^15^ decreasing total cholesterol^16^ and increasing natural immunity.^17^ In addition, oral administration of live *L. plantarum* K21 alleviates obesity induced by a high‐fat diet (HFD) in mouse models.^18^ Recently, to minimize the side effects of probiotics in vulnerable individuals, various studies were conducted to determine how postbiotics work and should be used.^19^ Postbiotics include functional bioactive compounds, such as metabolites, functional proteins, cell lysates and short‐chain fatty acids. Interestingly, the *Lactobacillus* extract can also inhibit adipogenesis in the mouse pre‐adipocyte 3T3‐L1 cell line.^20^ However, while ingesting *Lactobacillus* can help alleviate obesity and metabolic disorders, little is reported about the effects of consuming *Lactobacillus* postbiotics on obesity in animals.

In this study, we have confirmed that *L. plantarum* L‐14 extract inhibits the differentiation of 3T3‐L1 cells and human bone marrow mesenchymal stem cells (hBM‐MSCs) into mature adipocytes through the AMPK signalling pathway. The mouse HFD model studies have shown that periodic intake of L‐14 extract is effective in suppressing obesity, insulin resistance and hepatic steatosis. It has been found that exopolysaccharide (EPS) isolated from L‐14 extract is an effective substance for these anti‐adipogenic effects. In addition, the studies using C29 known as a Toll‐like receptor (TLR) 2 inhibitor have confirmed that EPS activates the AMPK signalling pathway by interacting with interacts with TLR2, and consequently inhibits lipid accumulation in 3T3‐L1 cells.

## 2 MATERIALS AND METHODS

### 2.1 Cell culture and general materials

We obtained the 3T3‐L1 cell line and hBM‐MSCs from the American Type Culture Collection (ATCC) and PromoCell, respectively. Details of cell culture and general materials are summarized in Appendix S1.

### 2.2 L‐14 extract preparation

We cultured *L. plantarum* L‐14 (KTCT13497BP) obtained from NeoRegen Biotech in de Man, Rogosa and Sharp (MRS) agar for 18 hours at 37°C, and the harvested L‐14 was sonicated on ice for 30 minutes using a sonicator. Details are summarized in Appendix S1.

### 2.3 Differentiation of 3T3‐L1 cells and hBM‐MSCs

We seeded 3T3‐L1 cells or hBM‐MSCs were seeded in 24‐well plates at a density of 1.0 × 10^5^ cells/well in the medium described in Appendix S1. Two days after confluence, the medium was changed with adipogenic induction medium (MDI). Mature adipocytes were analysed using Oil red O staining and TAG assay. Details are summarized in Appendix S1.

### 2.4 Cell viability assay

We seeded 3T3‐L1 cells in 96‐well plates at a density of 1.0 × 10^3^ cells/well. After 24 hours, we replaced the medium with different concentrations of L‐14 extract and maintained it for 4 days. Finally, we confirmed cell viability using the WST‐1 cell viability assay kit (Dongin LS).

### 2.5 Western blot analysis

Proteins were isolated using Cell Culture Lysis 1 × Reagent (Promega) containing a mixture of protease and phosphatase inhibitors (MedChemExpress). Protein signals on membranes were developed using electrochemiluminescence (ECL) Western blot analysis substrates (Daeillab Service) and analysed. Details are summarized in Appendix S1.

### 2.6 Real‐time quantitative polymerase chain reaction

We isolated messenger RNA (mRNA) from 3T3‐L1 cells, cultured it with L‐14 extract using the PureLink™ RNA minikit (Invitrogen) and reverse‐transcribed it into complementary DNA (cDNA) using a cDNA kit (Promega). Next, the cDNA was amplified using a TB green mix (TAKARA) using the StepOnePlus™ Real‐Time polymerase chain reaction (PCR) System (Applied Biosystems) and analysed. The primers used for quantitative reverse transcription PCR (qRT‐PCR) were designed as the listed sequences (Table S1).

### 2.7 Animals, diets and study design

All experiments on animals were performed in the accredited animal facility centre of the School of Dentistry, Seoul University, South Korea. All procedures involving animals were in compliance with the Korean legislation on animal experimentation, and the study was approved by the Institutional Animal Care and Use Committee of Seoul University (SNU‐180309‐1). Details of experiments on animals are summarized in Appendix S1.

### 2.8 Histology of mouse epididymal white adipose tissue and liver

We rinsed mouse epididymal white adipose tissue (eWAT) and liver twice with sterilized phosphate‐buffered saline (PBS) and fixed them in 4% paraformaldehyde (PFA) in PBS overnight. Next, the tissues were embedded into paraffin blocks, cut into 5‐μm‐thick slices and placed on adhesive microscope slides (Paul Marienfeld). Finally, the sections were analysed using haematoxylin and eosin (H&E) staining and immunohistochemistry (IHC). Details are summarized in Appendix S1.

### 2.9 Analysis of the effects of L‐14 extract and EPS on AMPK and TLR2 signalling pathways

We cultured 3T3‐L1 cells for 1 hour in starvation medium containing Dulbecco's modified Eagle's medium (DMEM) and 1% P/S. After starvation, we replaced the medium with normal medium containing 250 μmol/L 5‐aminoimidazole‐4‐carboxamide ribonucleotide (AICAR), 5 μmol/L Compound C (CC), and 50 μmol/L C29 and incubated the cells for 2 hours. Next, we induced the cells to differentiate into mature adipocytes in MDI with L‐14 extract or EPS, as described before, and analysed them. Details are summarized in Appendix S1.

### 2.10 EPS purification

We purified EPS using the ethanol precipitation method and identified it using the fast protein liquid chromatography (FPLC) system. Details are summarized in Appendix S1.

### 2.11 Statistical analysis

Statistical analysis was performed using SPSS Statistics version 25.0 (SPSS Inc.) and GraphPad Prism 5 (GraphPad). Data were presented as the mean ± standard deviation (SD). Data were analysed using analysis of variance (ANOVA). *P < .05, **P < .01 and ***P < .001 were considered statistically significant.

## 3 RESULTS

### 3.1 L‐14 extract inhibits 3T3‐L1 cell and hBM‐MSC differentiation into mature adipocytes

**FIGURE 1:**
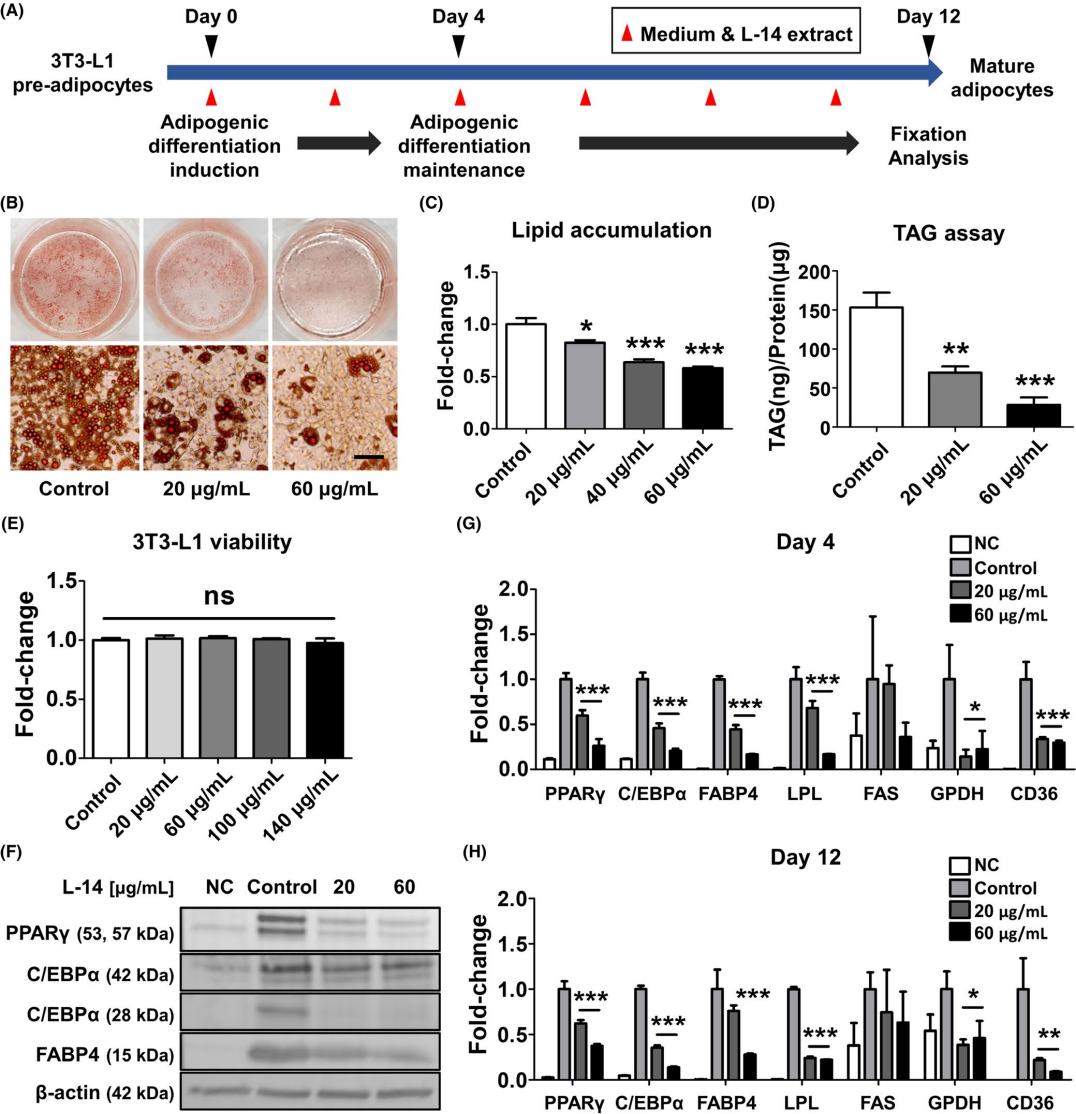
L‐14 extract inhibited differentiation of pre‐adipocyte 3T3‐L1 cells into mature adipocytes. A, The timeline of the experiments. After 4 d of adipogenic induction of pre‐adipocyte 3T3‐L1 cells, the adipogenic differentiation was maintained for 8 d. The fixation and analysis were performed at day 12. The cells were treated with various concentrations of L‐14 extract every two days, indicated by red arrows. B and C, Oil red O staining showed L‐14 extract inhibited lipid accumulation in 3T3‐L1 (scale bar = 100 μm). D, It was also confirmed that L‐14 extract suppressed triacylglycerol (TAG) storage through TAG assay. E, The viability of 3T3‐L1 was not affected by L‐14 extract. F, The protein expression of major adipogenesis markers (PPARγ, C/EBPα and FABP4) was decreased in 3T3‐L1 cultured with L‐14 extract for 12 d. G and H, The gene expression of adipogenic differentiation markers (PPARγ, C/EBPα, FABP4, LPS, FAS, GPDH and CD36) was also reduced by the treatment of L‐14 extract. Surprisingly, the markers were already changed by L‐14 extract in the early stage (days 0‐4) of adipogenic differentiation. The results are the means of at least three independent experiments (mean ± SD). *P < .05, **P < .01 and ***P < .001 vs the control group. NC means cells cultured in a normal medium as a negative control

We investigated the effects of L‐14 extract from *L*. *plantarum* on adipocyte adipogenesis in 3T3‐L1 cells. 3T3‐L1 cells were treated with 20 and 60 μg/mL of L‐14 extract every 2 days during adipogenic differentiation (Figure 1A). Oil red O staining showed that L‐14 extract inhibits lipid accumulation dose‐dependently (Figure 1B,C). In addition, L‐14 extract significantly decreased TAG storage in mature 3T3‐L1 cells (Figure 1D). However, L‐14 extract did not affect the cell viability of 3T3‐L1 cells, indicating that the inhibitory effect on adipocyte differentiation is not due to the cytotoxicity of L‐14 extract (Figure 1E). Western blot analysis showed that L‐14 extract decreases the major adipogenesis markers peroxisome proliferator‐activated receptor gamma (PPARγ), CCAAT‐enhancer‐binding protein alpha (C/EBPα) and fatty acid‐binding protein 4 (FABP4) (Figure 1F). In addition, L‐14 extract significantly decreased the gene expression of adipogenic differentiation markers in 3T3‐L1 cells, including PPARγ, C/EBPα, FABP4, lipoprotein lipase (LPL), fatty acid synthase (FAS), glycerol‐3‐phosphate dehydrogenase (GPDH) and CD36 (Figure 1G,H). Interestingly, L‐14 extract had already decreased these markers in the early stage (days 0‒4) of adipogenic differentiation. These results showed that the L‐14 extract‐induced adipogenesis inhibition is due to a decrease in the expression of adipogenic factors in the early stage of differentiation.

### 3.2 Periodic oral intake of L‐14 extract decreases weight gain and expression of pro‐inflammatory markers in the HFD mouse model

**FIGURE 2:**
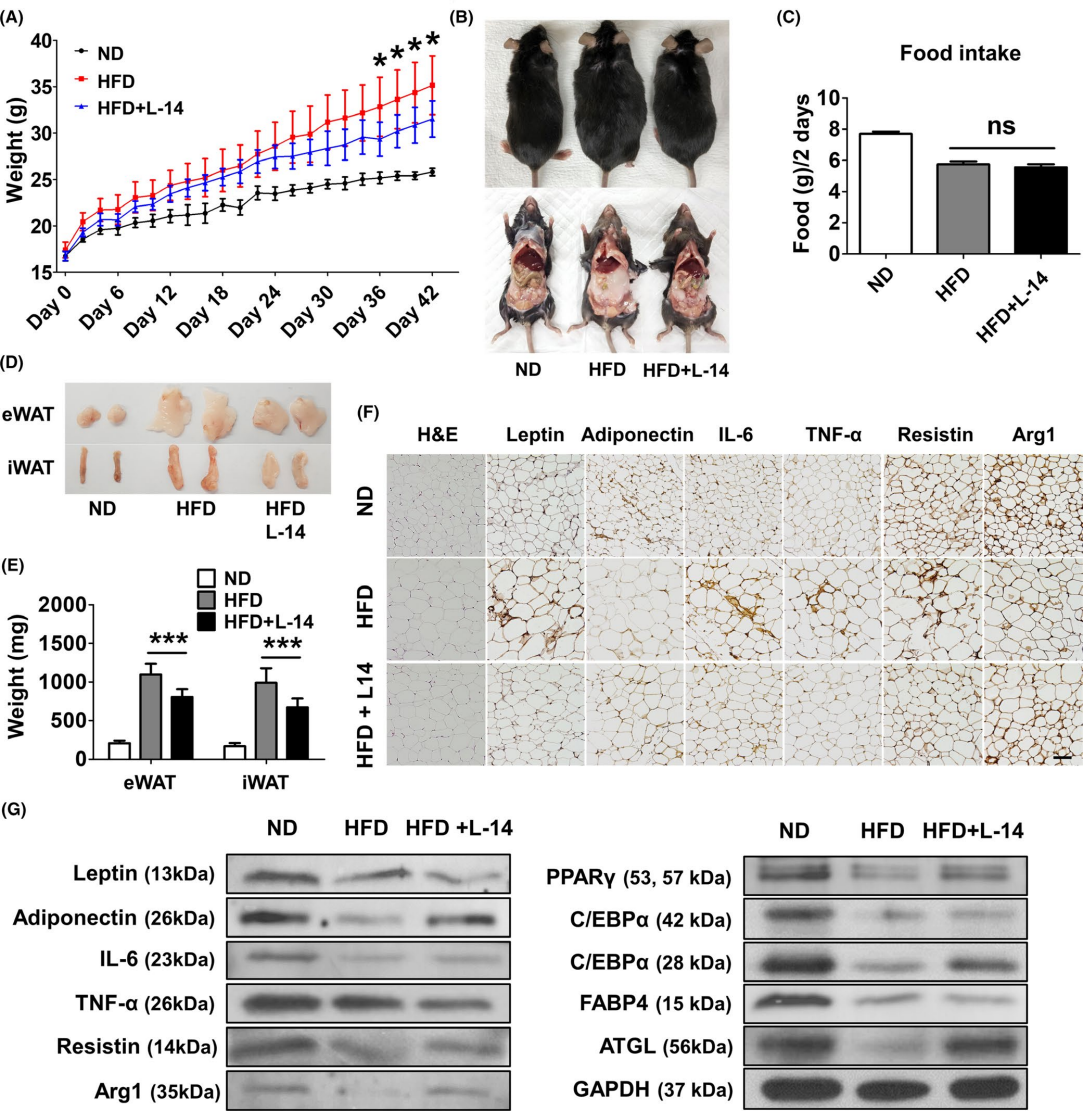
Periodic intake of L‐14 extract reduced weight gain and expression of pro‐inflammatory markers in the high‐fat diet (HFD) model. C57BL/6J mice were randomly divided into three groups: normal diet (ND, n = 6), HFD (n = 7) and L‐14 (HFD with L‐14 extract, n = 8). L‐14 extract (500 mg/kg) or PBS as control was orally administrated to HFD‐feeding mice every two days for 7 wk. A and B, The average weight of L‐14 mice was significantly different from that of HFD mice after 36 d. C, There was no difference of food intake between HFD and HFD + L‐14 mice. D and E, The consumption of the L‐14 reduces fat mass of epididymal white adipose tissue (eWAT) and inguinal white adipose tissue. F and G, H&E staining showed L‐14 mice had much smaller size of adipocytes in eWAT than HFD mice. Also, the expression of the pro‐inflammatory markers (leptin, IL‐6, TNF‐α and resistin) was decreased while that of the anti‐inflammatory markers (adiponectin and Arg1) was increased by the L‐14 consumption (scale bar = 100 μm). The results are presented as the average of the measurements in each group (Mean ± SD). *P < .05 and ***P < .001 vs the HFD group

To confirm the effects of oral intake of L‐14 extract in vivo, we randomly divided C57BL/6J mice into three groups (normal diet (ND), HFD and HFD + L ‐14) and orally administrated 500 mg/kg of L‐14 extract or PBS (control) to HFD‐feeding mice every 2 days for 7 weeks. The weight of the HFD + L‐14 group (31.51 ± 1.96 g) was significantly different from that of the HFD group (35.14 ± 3.18 g) (Figure 2A,B). We observed no significant change in the food intake following L‐14 administration (Figure 2C). The oral intake of L‐14 extract significantly decreased the fat mass of eWAT from 1.10 ± 0.14 to 0.81 ± 0.10 g and of inguinal white adipose tissue (iWAT) from 0.99 ± 0.19 to 0.67 ± 0.12 g (Figure 2D,E). In addition, H&E staining showed that the adipocyte size of eWAT was much smaller in the HFD + L‐14 group compared to the HFD group (Figure 2F). L‐14 extract also decreased the expression of pro‐inflammation markers, such as leptin, interleukin‐6 (IL‐6), tumour necrosis factor alpha (TNF‐α) and resistin, and increased the expression of anti‐inflammation markers, such as adiponectin and arginase 1 (Arg1) (Figure 2F,G).

### 3.3 L‐14 extract decreases insulin resistance marker and steatohepatitis

**FIGURE 3:**
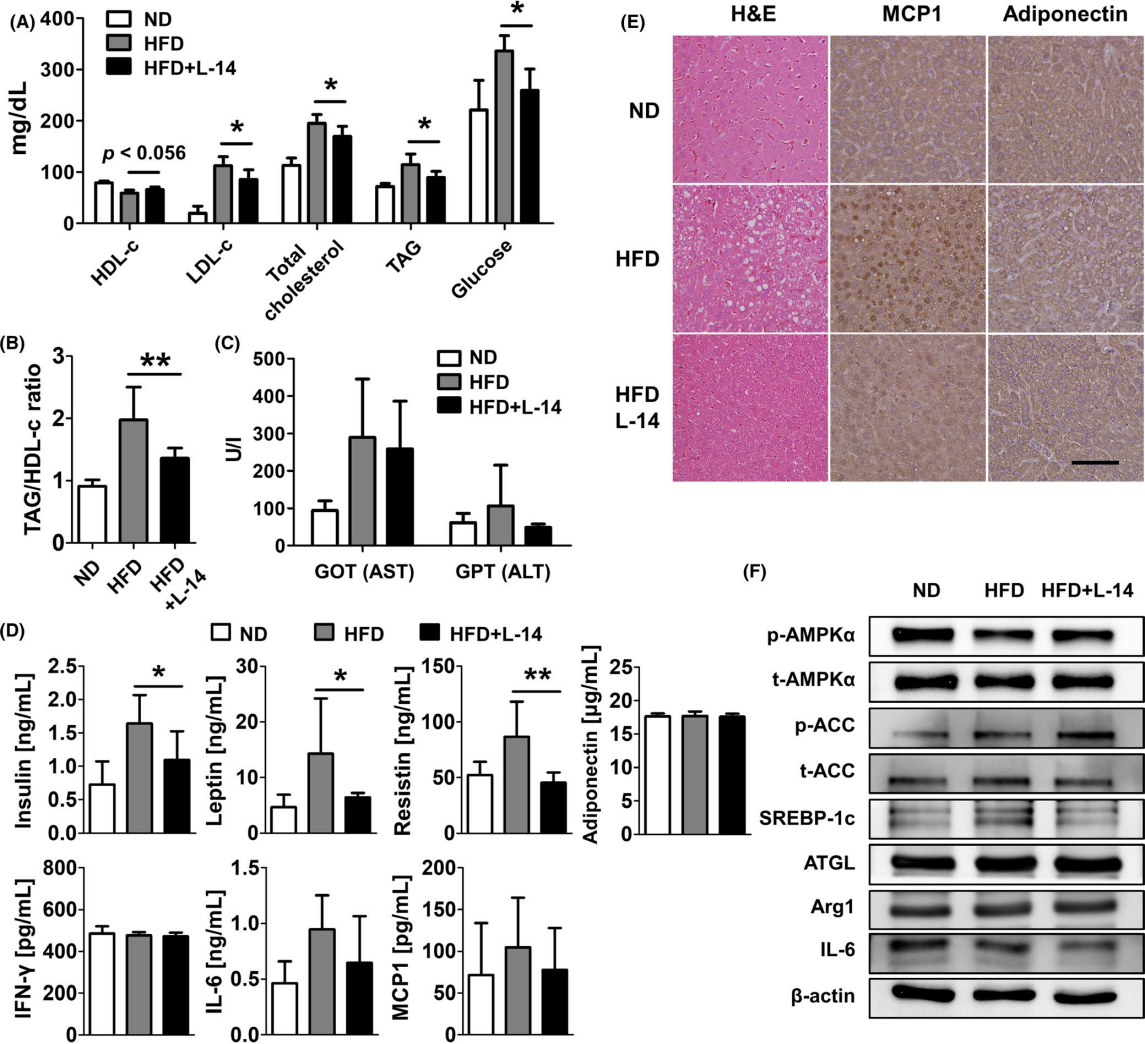
L‐14 extract intake reduced insulin resistance marker and hepatic steatosis. A, The marked decrease in LDL‐c and TAG levels and parallel increase in HDL‐c (*P* < .056) were detected in the serum of L‐14‐consuming mice compared to HFD‐feeding mice. B, An insulin resistance marker in serum, TAG/ HDL‐c was significantly reduced by L‐14 intake. C and D, Fasting glucose, fasting insulin, leptin and resistin of the L‐14 group were decreased compared to the HFD group, whereas GOT (AST), GPT (ALT), adiponectin, IFN‐γ, IL‐6 and MCP1 were not significantly changed. E, The HFD‐induced hepatic steatosis and MCP1 were attenuated by L‐14 extract (scale bar = 100 μm). F, The AMPK signalling pathway was activated by the L‐14 while the expression of IL‐6 was inhibited despite no change of Arg1 and ATGL expressions. The results are presented as the average of the measurements in each group (Mean ± SD). *P < .05 and ***P < .001 vs the HFD group

We confirmed the effects of periodic oral intake of L‐14 extract via a blood chemistry test and enzyme‐linked immunosorbent assay (ELISA). Serum low‐density lipoprotein cholesterol (LDL‐c) and TAG levels significantly decreased, while serum high‐density lipoprotein cholesterol (HDL‐c) levels significantly increased (*P* < .056) in the HFD + L‐14 group compared to the HFD group (Figure 3A). L‐14 extract consistently decreased total cholesterol and also decreased the TAG/HDL‐c ratio (an insulin resistance marker) (Figure 3B). In addition, L‐14 extract decreased fasting glucose, fasting insulin, leptin and resistin levels, while aspartate transaminase (AST), alanine transaminase (ALT), adiponectin, interferon gamma (IFN‐γ), IL‐6 and monocyte chemoattractant protein 1 (MCP1) levels did not change significantly (Figure 3C,D).

To confirm the effects of L‐14 extract on the liver, we histologically analysed liver tissue samples isolated from the HFD group. H&E staining showed that L‐14 extract attenuates steatohepatitis induced by an HFD (Figure 3E). In addition, L‐14 extract inhibited the expression of MCP1 and IL‐6 was but activated the AMPK signalling pathway (Figure 3F). Although we observed no significant changes of Arg1 and adipose triglyceride lipase (ATGL) expression involved with lipolysis, L‐14 extract decreased the IL‐6 expression in the liver.

### 3.4 L‐14 extract inhibits adipogenesis in 3T3‐L1 cells by upregulating the AMPK signalling pathway in the early stage of adipogenic differentiation

**FIGURE 4:**
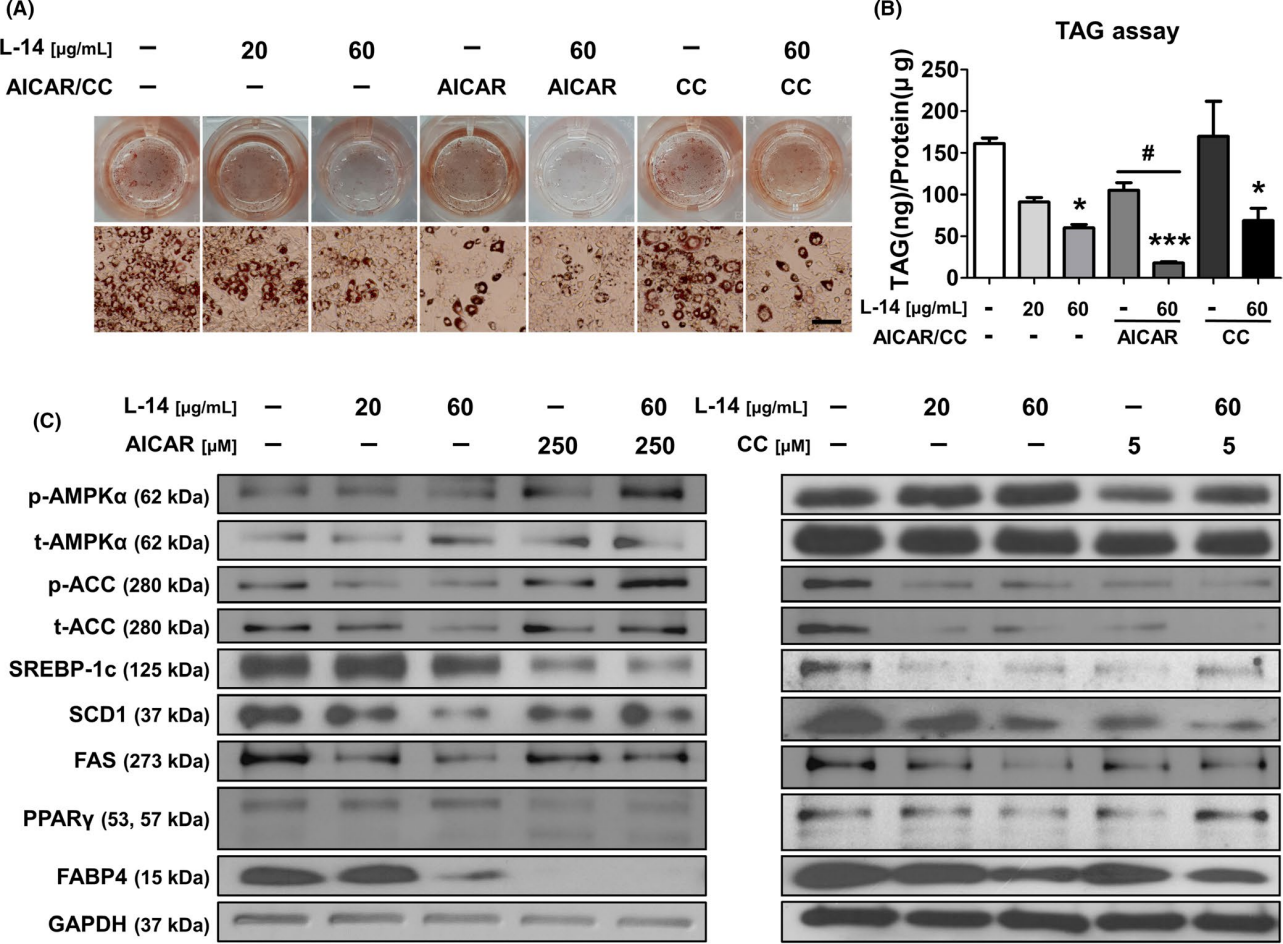
L‐14 extract inhibited adipogenesis in mouse pre‐adipocytes by upregulating AMPK signalling pathway in the early stage of adipogenic differentiation. A and B, L‐14 extract reduced the lipid contents in 3T3‐L1 under AMPK‐activated or even inhibited conditions (scale bar = 100 μm). C, In early stage (days 0‐4) of adipogenic differentiation, L‐14 extract inhibited the expression of AMPK signalling pathway‐related markers in synergetic effect with AICAR while recovering the phosphorylation of AMPKα which inhibited by Compound C. These results showed that L‐14 extract inhibited the differentiation of mouse pre‐adipocytes into mature adipocytes by upregulating AMPK signalling pathway in the early stage of adipogenic differentiation. The results are the means of at least three independent experiments (mean ± SD). *P < .05 and ***P < .001 vs the control group and ^#^ *P* < .05 vs the only AICAR‐treated group

To determine whether the AMPK signalling pathway mediates the inhibitory effect of L‐14 extract on adipogenic differentiation, 3T3‐L1 cells grown in the AMPK activator AICAR and the AMPK inhibitor CC were treated with L‐14 extract. AICAR significantly decreased the lipid content of 3T3‐L1 cells after adipogenic differentiation, and additional L‐14 extract administration enhanced the inhibitory effect of AICAR. CC did not affect lipid accumulation in 3T3‐L1 cells, and L‐14 extract decreased TAG levels even under AMPK‐inhibited conditions (Figure 4A,B). These results showed that the AMPK signalling pathway mediates the anti‐adipogenic effects of L‐14 extract.

**FIGURE 5:**
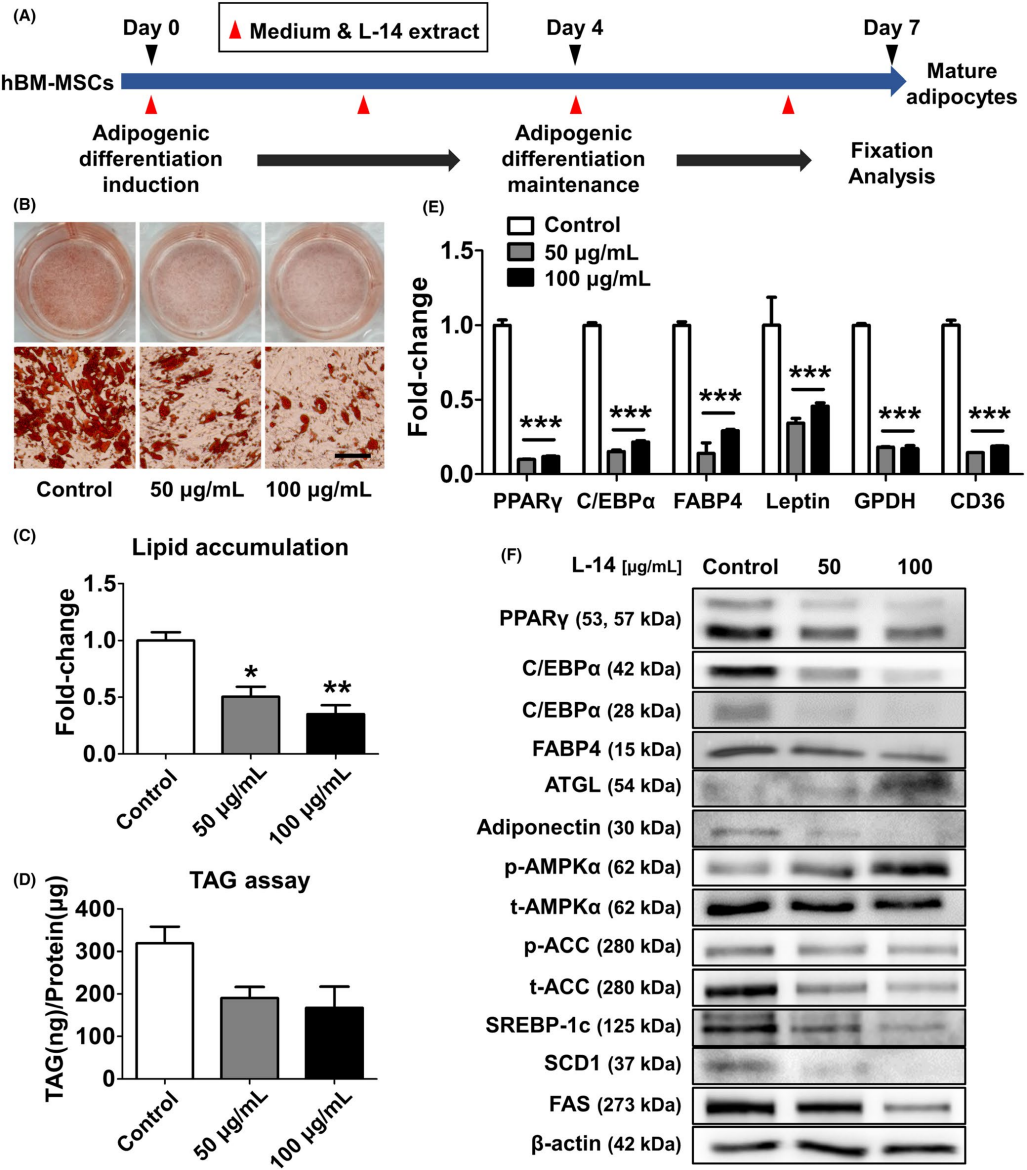
L‐14 extract inhibited differentiation of human bone marrow mesenchymal stem cells (BM‐MSCs). A, The timeline of the experiment using hBM‐MSCs. After 4 d of adipogenic induction of hBM‐MSCs, the adipogenic differentiation was maintained for 3 d. The fixation and analysis were performed at day 7. The cells were treated with 50 and 100 μg/mL of L‐14 extract every two days, indicated by red arrows. B and C, Oil red O staining showed L‐14 extract inhibited lipid accumulation in hBM‐MSCs (scale bar = 100 μm). D, It was also confirmed that L‐14 extract suppressed TAG storage through TAG assay. E, The gene expression of adipogenic differentiation markers (PPARγ, C/EBPα, FABP4, leptin, GPDH and CD36) was also reduced by L‐14 extract. F, The protein expression of major adipogenesis markers and adiponectin was decreased and that of AMPK signalling pathway markers was increased in hBM‐MSCs cultured with L‐14 extract for 4 d. These results showed that L‐14 extract inhibited the differentiation of hBM‐MSCs into mature adipocytes by upregulating AMPK signalling pathway. The results are the means of at least three independent experiments (mean ± SD). *P < .05, **P < .01 and ***P < .001 vs the control group

To determine whether L‐14 extract can activate the AMPK signalling pathway in the early stage (days 0‒4) of adipogenic differentiation, we analysed the related markers by Western blot analysis. AICAR and L‐14 extract synergistically activated the AMPK signalling pathway within 4 days, significantly decreasing the expression of adipogenesis markers such as PPARγ and FABP4 (Figure 4C). In addition, L‐14 extract increased AMPKα phosphorylation, which was inhibited by CC. Simultaneous AICAR and L‐14 extract administration synergistically inhibited TAG accumulation. Interestingly, L‐14 extract also inhibited the adipogenesis of hBM‐MSCs by upregulating the AMPK signalling pathway in the early stage (day 4) of adipogenic differentiation (Figure 5). These results showed that L‐14 extract inhibits the differentiation of mouse 3T3‐L1 cells and hBM‐MSCs into mature adipocytes by upregulating the AMPK signalling pathway in the early stage of adipogenic differentiation.

### 3.5 EPS isolated from L‐14 extract regulates adipogenic differentiation through the TLR2 signalling pathway

**FIGURE 6:**
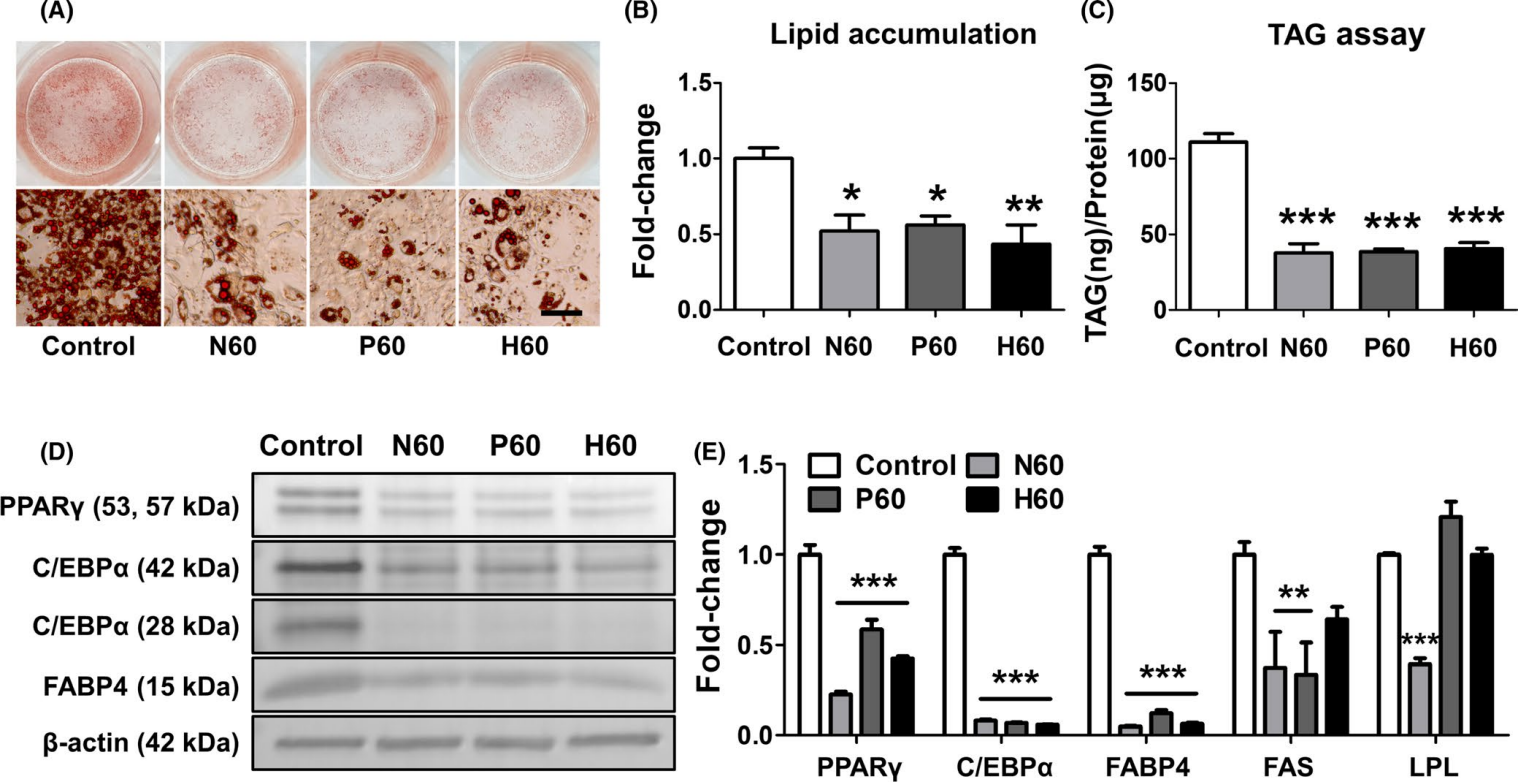
Anti‐adipogenesis effect of L‐14 extract (N60) was not affected by changes of pH (P60) or incubation in harsh thermal conditions (H60). A‐C, Although L‐14 extract was heated to 90°C for 30 min or the pH was changed to neutral, the adipogenesis inhibitory effect was not changed, indicating that the effective molecules with the effect were stable (scale bar = 100 μm). D and E, The inhibitory effect of major adipogenesis markers (PPARγ, C/EBPα and FABP4) by L‐14 extract was not significantly changed by heating and pH changes. However, the gene expression of FAS was not reduced by H60, and the gene expression of LPL was not affected by P60 and H60. The results are the means of at least three independent experiments (mean ± SD). *P < .05, **P < .01 and ***P < .001 vs the control group

Although our results confirmed that L‐14 extract inhibits the differentiation of 3T3‐L1 cells into mature adipocytes, we tried to identify the key molecule with adipogenesis inhibitory effects in L‐14 extract. The effects of L‐14 extract did not change by a change in pH or by incubation in harsh thermal conditions, indicating that the key molecule with adipogenesis inhibitory effects is stable to heat (Figure 6).

**FIGURE 7:**
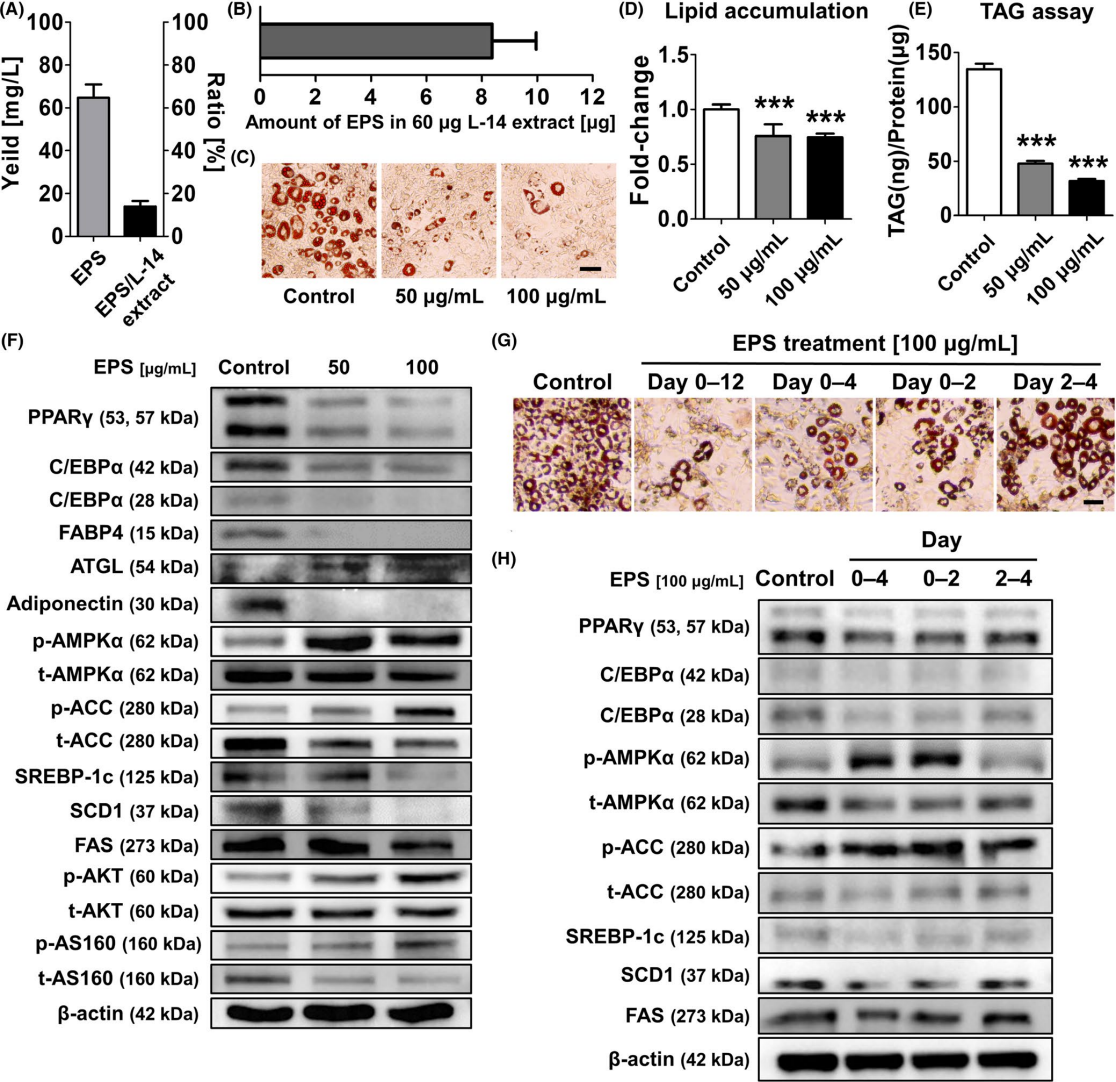
Exopolysaccharide inhibited lipid accumulation by activating the AMPK signalling pathway in the early stage of adipocyte differentiation. A, The results showed that 13.95 ± 2.30% (n = 4) of L‐14 extract was composed of EPS. B, Thus, 60 ug of L‐14 extract contained 8.37 ± 1.38 μg of EPS. C‐E, At higher concentrations than L‐14 extract, EPS significantly inhibited adipogenesis of 3T3‐L1. F, The treatment of EPS induced the upregulation of AMPK signalling pathway and downregulation of adipogenesis markers and adiponectin. Also, the phosphorylation of AKT and AS160 was upregulated after EPS treatment. G, The treatment with EPS at early stages (days 0‐4 and 0‐2) remarkably inhibited lipid accumulation. H, The treatment with EPS (days 0‐4 and 0‐2) significantly upregulated the AMPK signalling pathway compared to day 2‐4 treatment. These results indicated that EPS plays a critical role in inhibiting lipid accumulation at the beginning stage of adipogenic differentiation. The results are the means of at least three independent experiments (mean ± SD). ***P < .001 vs the control group

Next, we isolated EPS from L‐14 extract and confirmed it to be a homogeneous polysaccharide through FPLC in the previous studies. We also confirmed that 13.95 ± 2.30% (n = 4) of L‐14 extract was composed of EPS (Figure 7A), which indicated that the amount of EPS in 60 μg L‐14 extract was 8.37 ± 1.38 μg (Figure 7B). To confirm whether EPS inhibits lipid accumulation, we cultured 3T3‐L1 cells with EPS in MDI for 12 days. Although higher concentrations of EPS were required, EPS showed adipogenesis inhibitory effects (Figure 7C‐E). In addition, Western blot analysis showed that EPS upregulates the AMPK signalling pathway and downregulates the expression of adipogenesis markers and adiponectin (Figure 7F). Interestingly, EPS also increased the phosphorylation of protein kinase B (AKT) and the AKT substrate of 160 kDa (AS160), which are related to glucose uptake in cells. We also investigated the pivotal stage of adipogenesis, particularly affected by EPS treatment. The treatment with EPS only at early stages (days 0‐4 and 0‐2) remarkably inhibited lipid accumulation (Figure 7G). The results also confirmed that the treatment with EPS (days 0‐4 and 0‐2) significantly upregulated the AMPK signalling pathway compared to day 2‐4 treatment, suggesting that EPS plays a critical role in inhibiting lipid accumulation at the beginning stage of adipogenic differentiation (Figure 7H).

**FIGURE 8:**
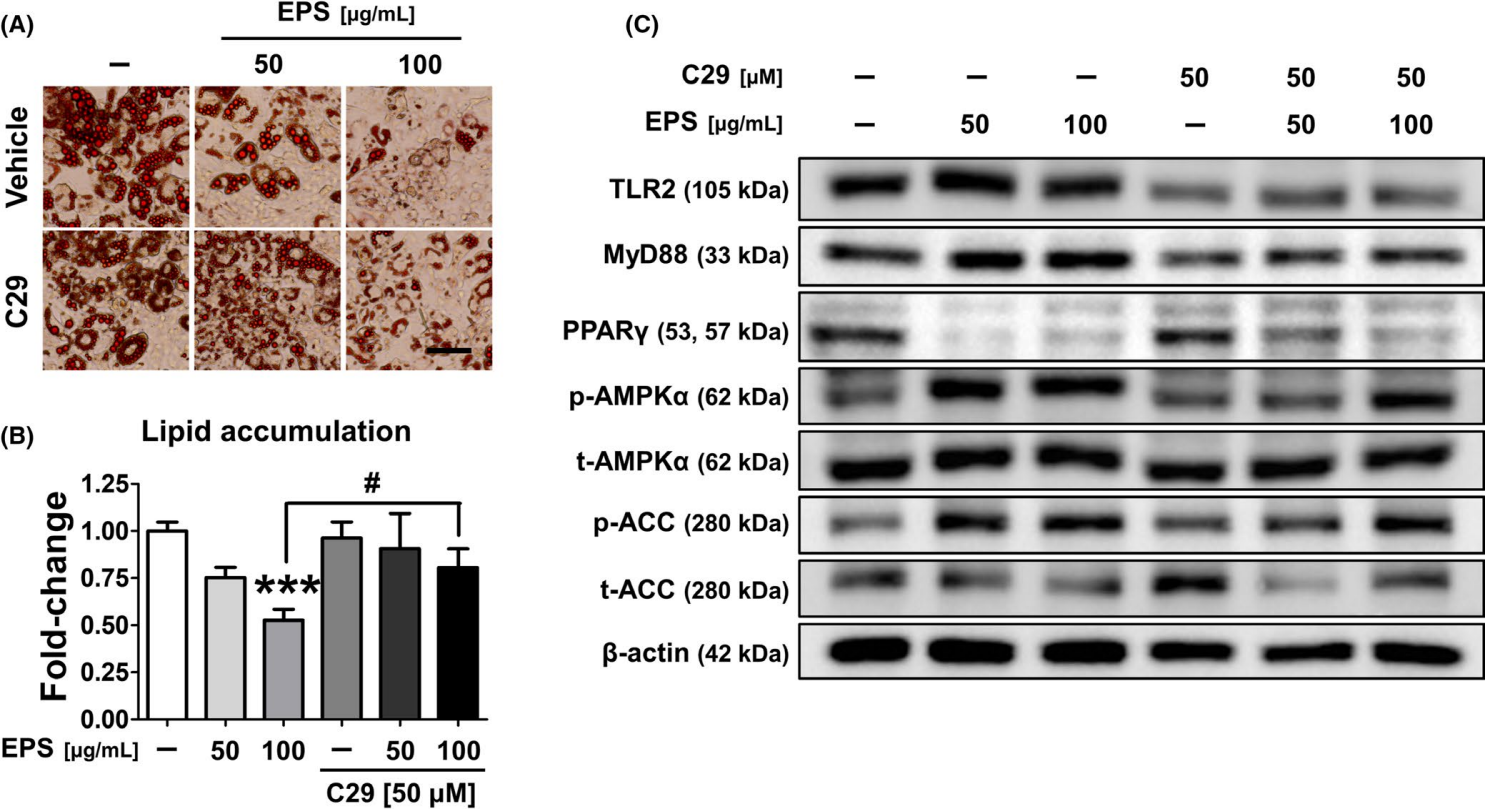
Exopolysaccharide isolated from L‐14 extract regulated adipogenic differentiation through the TLR2 pathway. A and B, Although the TLR2 inhibitor C29 had no effect on adipogenesis, it reduced the adipogenesis inhibitory effect of EPS (scale bar = 100 μm). C, The activation of AMPK signalling pathway by EPS was inhibited, when the expression of TLR2 and MyD88 was inhibited by C29, suggesting that EPS activates the AMPK signalling pathway by interacting with TLR2, and consequently inhibits adipogenesis. The results are the means of at least three independent experiments (mean ± SD). ***P < .001 vs the control group and ^#^ *P* < .05 vs the only EPS‐treated group

To determine which receptors in cells interacted with EPS, we performed further studies using the TLR2 inhibitor C29. C29 did not affect lipid accumulation, but the adipogenesis inhibitory effects of EPS significantly decreased in the C29‐treated group (Figure 8A,B). The results showed that activation of the AMPK signalling pathway by EPS was inhibited in the early stage (day 4) of adipogenic differentiation, when TLR2 and myeloid differentiation primary response 88 (MyD88) expression is inhibited by C29, indicating that EPS activates the AMPK signalling pathway by interacting with TLR2, consequently inhibiting adipogenesis (Figure 8C).

## 4 DISCUSSION

The increasing prevalence of obesity is a public health issue that must be solved urgently, and the global concern about obesity‐associated metabolic syndrome is growing. Since systemic oxidative stress induced by fat accumulation causes obesity‐associated metabolic syndrome, obese people have a higher mortality rate compared to healthy people.^21^, ^22^ Over the past few decades, many studies have described characteristic differences of the gut microbiota between lean and obese individuals.^23^ There have been attempts to prevent and/or treat obesity by improving the gut microbiota using probiotics, such as *Lactobacillus* and *Bifidobacterium* strains. Different *Lactobacillus* strains have distinct effects on anti‐adipogenesis in 3T3‐L1 cells, and L‐14 extract inhibits TAG accumulation more than other extracts do.^24^ Interestingly, our preliminary results have shown that the inhibitory effects of L‐14 extract on adipocyte differentiation even fluctuate depending on the subspecies of *L. plantarum*. *L. plantarum* L‐14 in traditional fermented food has beneficial effects on human health, and L‐14 extract shows anti‐adipogenesis activity without affecting cell viability (Figure 1B‐E). Several studies have shown that the fate of 3T3‐L1 cells is determined at a very early stage.^25^, ^26^ L‐14 extract acts in the early stage of adipocyte differentiation and, consequently, inhibits lipid accumulation (Figures 1G and 4C).

Double‐blind randomized clinical trials have shown that periodic oral intake of probiotics might decrease fat mass and/or body weight, although a risk of bias existed.^27^ However, the effects of oral intake of *Lactobacillus* extract have rarely been studied, although intraperitoneal injection of *Lactobacillus* extract for 2 weeks significantly decreases the systemic fat mass.^24^ Oral intake of L‐14 extract has both systemic beneficial effects and anti‐obesity effects. In this study, after ~5 weeks, there was a significant difference in body weight between the HFD group and the HFD + L‐14 group, which was due to the difference in fat mass (Figure 2A‐E). Interestingly, L‐14 extract increased PPARγ and C/EBPα expressions in eWAT under HFD conditions. Previous studies with PPARγ agonists have shown that TNF‐α downregulation upregulates PPARγ expression, which would improve insulin sensitivity.^28^ The interaction between adipose tissue and adipose tissue macrophage (ATM) polarization plays a critical role in obesity progression.^29^ Chronic exposure to an HFD remodels the adipose tissue by infiltration of monocytes and differentiation into M1 ATMs and increases the local secretion of pro‐inflammatory cytokines, such as IL‐6 and TNF‐α, in stressful environments, such as hypoxia, increased reactive oxygen species and adipocyte necrosis. In contrast, the alternative M2 ATMs maintained by PPARγ and Krüppel‐like factor 4 produce anti‐inflammatory cytokines, such as Arg1. Oral intake of L‐14 extract could inhibit the dysfunction of adipose tissue by inhibiting hypertrophic adipocytes and polarization to M1 ATMs.^30^ In addition, L‐14 extract decreases fat accumulation and MCP1 expression in the liver (Figure 3E,F). HFD‐induced liver injury causes Kupffer cells to present the M1 phenotype and increases intrahepatic MCP1 expression. The infiltrating macrophages recruited by MCP1 produce TNF‐α, which could again increase MCP1 expression.^31^ This positive feedback loop might further exacerbate non‐alcoholic fatty liver disease, triggering non‐alcoholic steatohepatitis or liver failure. Inflammatory cytokines secreted by M1 ATMs in steatohepatitis increase hepatic cholesterologenesis and TAG production, leading to regulatory failure of lipid metabolism and homeostasis.

Obesity is clearly associated with the levels of hormones, such as leptin, adiponectin and resistin secreted from adipose tissue.^32^ These hormones, together with various regulatory molecules, play a crucial role in energy metabolism and body weight homeostasis. Therefore, an imbalance in the regulation of these hormones is a major risk factor for the pathological process of obesity. Especially, maintenance of leptin overexpression leads to leptin resistance via impairment of the leptin signalling pathway, which impairs the regulatory capacity of satiety and metabolism related to the AMPK signalling pathway.^33^ In this study, leptin expression was higher in the HFD group compared to the HFD + L‐14 group, and there was no between‐group difference in food intake, indicating that the physiological regulation by leptin is impaired in the HFD group (Figures 2C and 3C). In other words, periodic oral intake of L‐14 extract could ameliorate leptin functional failure induced by an HFD. Adiponectin increases lipid accumulation in adipocytes, inhibiting ectopic lipid accumulation, and enhances insulin sensitivity in vivo.^34^ In addition, adiponectin is regulated by PPARγ in adipocytes,^35^, ^36^ and consistent with previous studies, adiponectin expression in vitro and in vivo is regulated in the same manner as PPARγ by L‐14 extract or EPS (Figures 2G and 7E). L‐14 extract significantly decreases resistin (an inflammation and insulin resistance marker) and serum LDL‐c, total cholesterol, and TAG levels. Accumulating evidence has shown that the TAG/HDL‐c ratio is correlated with generally recognized methods of defining insulin resistance.^37^ AKT‐mediated phosphorylation of Rab GTPase‐activating protein AS160 can allow glucose transporter type 4 (GLUT4) translocation to the plasma membrane and subsequently enhance glucose uptake.^38^ Since activated AMPK can also induce AS160 phosphorylation,^39^ EPS can increase the glucose uptake of adipocytes through the AS160‐mediated pathway (Figure 7F) and might be used as a potential substance to treat insulin resistance or type 2 diabetes.

L‐14 inhibits the differentiation of 3T3‐L1 cells and hBM‐MSCs into mature adipocytes, which is mediated by the AMPK signalling pathway (Figures 4 and 5). Since the AMPK signalling pathway plays an important role in the early stage of adipocyte differentiation,^40^ our experiments using AICAR and CC (AMPK activator and inhibitor, respectively) confirmed that L‐14 extract can inhibit lipid accumulation by regulating the downstream targets of AMPK, such as acetyl‐CoA carboxylase (ACC), sterol regulatory element‐binding protein 1c (SREBP‐1c), stearoyl‐CoA desaturase 1 (SCD1) and FAS at the early stage of adipogenic differentiation (Figure 4C). Interestingly, although the AMPK signalling pathway is inhibited by CC at the protein level, TAG accumulation does not change. However, L‐14 extract recovers the AMPKa and ACC phosphorylation inhibited by CC. In addition, the adipogenesis inhibitory effect is not caused by pH, and the molecule is stable to heat (Figure 6). The EPS of *Lactobacillus rhamnosus GG* can inhibit the adipogenesis of 3T3‐L1 cells.^24^ In a previous study, we isolated the EPS from L‐14 extract and confirmed that it can inhibit the inflammatory response by LPS in mouse RAW 264.7 cells.^41^ We also confirmed the properties of EPS through various physicochemical analysis. It was shown that EPS has a single symmetrical peak through FPLC and consists of glucose using thin layer chromatography. Studies using Fourier‐transform infrared spectroscopy also showed that EPS has absorption bands at 1032.58 cm^‐1^ and 812.28 cm^‐1^, indicating a presence of polysaccharide and side groups of carbohydrates, respectively. These results indicated that EPS was a homogeneous polysaccharide composed of glucose. In addition, through Gel permeation chromatography, a weight average molecular weight (Mw), a number average molecular weight (Mn) and a size average molecular weight (Mz) of EPS were determined to be 7.57 × 10^4^ Da, 1.84 × 10^4^ and 3.74 × 10^5^, respectively. In this study, the EPS of L‐14 extract inhibited lipid accumulation through the AMPK signalling pathway in the same way as L‐14 extract. The previous studies have shown that the drug treatment at early adipogenic stage (days 0‐2) markedly inhibited adipogenesis, and these inhibitory effects were similar to that of continuous treatment (days 0‐8).^26^ Our results also showed that EPS activated the AMPK signalling pathway in the early stage of adipogenic differentiation, and especially, the initiating stage of differentiation (days 0‐2) was the most critical stage in the inhibition of lipid accumulation by EPS (Figure 7G,H). However, a higher concentration of EPS was required to reduce adipogenesis compared to L‐14 extract. The concentrations of L‐14 extract and EPS which were required to reduce TAG accumulation below 40% were 60 μg/mL and 50 μg/mL, respectively (Figures 1D and 7E). According to the data in Figure 7B, 60 μg of L‐14 extract contains 8.37 ± 1.38 μg of EPS. Therefore, even though the amount of EPS in 60 μg of L‐14 extract was approximately 5.97 times less than 50 μg of EPS, it had the effect of inhibiting TAG accumulation similar to that of 50 μg/mL EPS. These results indicated that in addition to EPS, there are other key molecules in L‐14 extract that inhibit adipogenesis of 3T3‐L1 cells. Although we have not identified other molecules that affect the adipogenesis of 3T3‐L1 cells, the effect of EPS on the AMPK signalling pathway is the same as or more evident than that of L‐14 extract (Figures 4C and 7E), indicating that EPS is the key molecule that produces the adipogenesis inhibitory effects of L‐14 extract.

A previous clinical study has shown that TLR2, TLR4 and MyD88 expressions are significantly elevated in the adipose tissue of obese individuals.^42^ Especially, MyD88 deficiency causes weight gain, impaired glucose homeostasis and increased expression of pro‐inflammatory cytokines, indicating that regulating MyD88 expression can be a potential approach to controlling metabolic disorders and inflammatory diseases.^43^ Our experiments using the TLR2 inhibitor showed that EPS regulates AMPK and PPARγ signalling‐dependent adipogenesis via TLR2 and MyD88 signalling pathways in the early stage (Figure 8A‐C). However, C29 treatment without EPS does not significantly affect the differentiation of 3T3‐L1 cells, indicating that the TLR2 and MyD88 downregulation does not directly regulate lipid accumulation. Therefore, further studies are required in order to determine how EPS interacts with TLR2 and regulates the AMPK signalling pathway.

**FIGURE 9:**
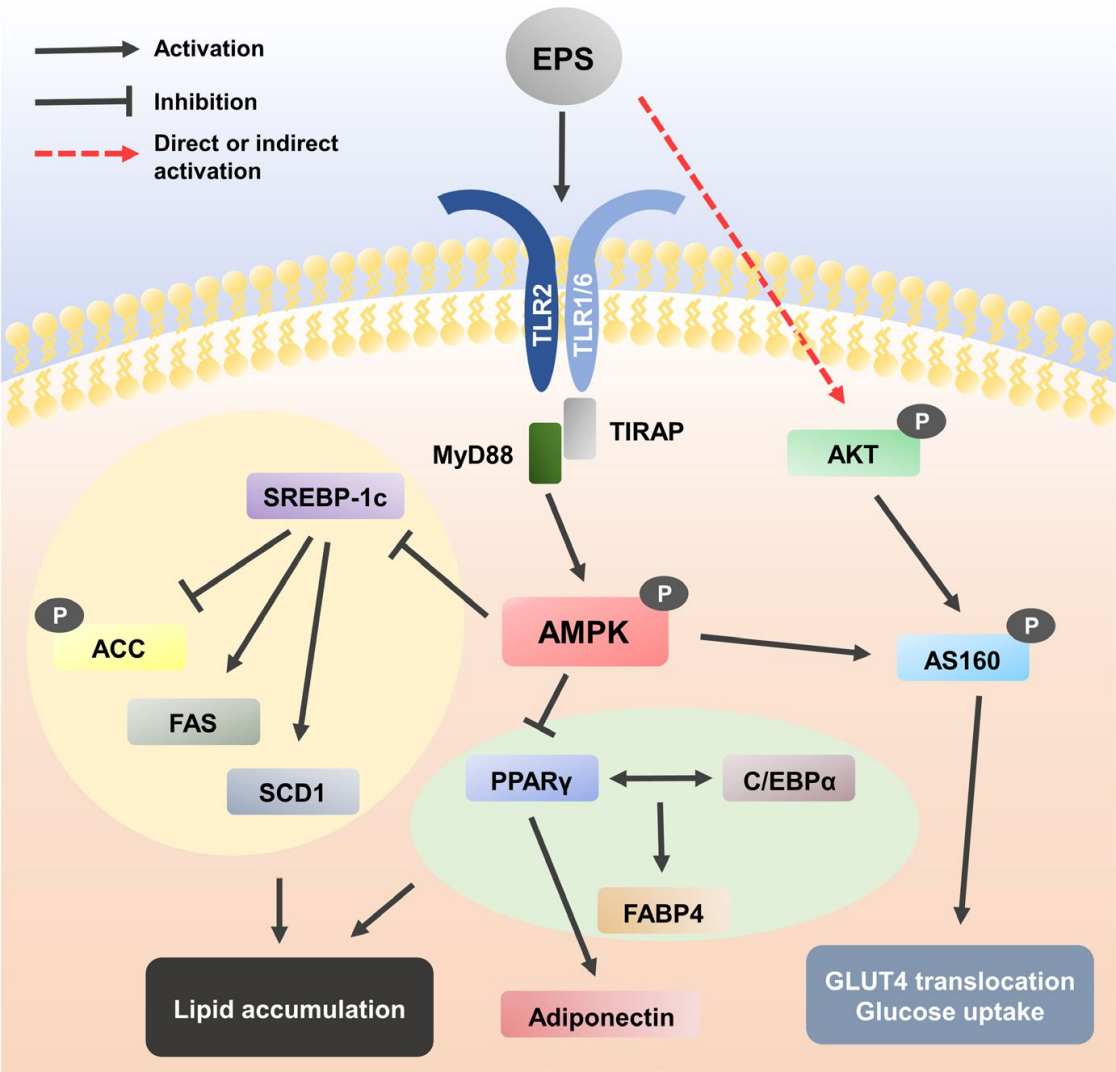
Eexopolysaccharide regulates lipid accumulation and glucose uptake via TLR2 and AMPK signalling pathway. When EPS binds to TLR2 in the cell membrane, MyD88, a downstream adaptor protein of TLR, initiating AMPK signalling cascades. The phosphorylated AMPKα inactivates SREBP‐1c, ACC, FAS and SCD1 by phosphorylation. In addition, the activated AMPKα inhibits the expression of core adipogenesis markers including PPARγ, C/EBPα and FABP4. The reduced PPARγ expression decreases the expression of adiponectin. These changes eventually reduce lipid accumulation in adipocytes. Also, EPS induces phosphorylation of AKT through a direct or indirect pathway and the activated AKT and AMPKα phosphorylates AS160. The activated AS160 can allow GLUT4 translocation to plasma membrane and subsequently enhance glucose uptake

In conclusion, L‐14 extract from Lactobacillus significantly inhibits the differentiation of mouse 3T3‐L1 cells and hBM‐MSCs to mature adipocytes via AMPK and PPARγ signalling pathways and alleviates systemic inflammation and obesity in vivo in an HFD mouse model. In addition, the EPS isolated from L‐14 extract is the key molecule that has these effects, and it is mediated via TLR2 and MyD88 signalling pathways (Figure 9). Therefore, although further studies are required in order to confirm that EPS has the same effects as L‐14 extract on the HFD mouse model without side effects, EPS can be used as the potential substance of postbiotics for the purpose of preventing and treating metabolic disorders, such as obesity and type 2 diabetes.

## CONFLICT OF INTEREST

The authors declare no competing financial interest.

## AUTHOR CONTRIBUTIONS

JL and SP designed the experiments under the supervision of JS and SR. JL and JP wrote the manuscript. JL, NO and MK performed the experiments and analysed the data. JL and SP performed the literature research. JL designed the research template. All authors have read and agreed to the published version of the manuscript.

## Supporting information

Supplementary Material

## Data Availability

The data that support the findings of this study are available from the corresponding author upon reasonable request.
