# Supplementary material for: Oral intake of Lactobacillus plantarum L‐14 extract alleviates TLR2‐ and AMPK‐mediated obesity‐associated disorders in high‐fat‐diet‐induced obese C57BL/6J mice
Source: Cell Prolif. 2021 Apr 8;54(6):e13039. doi: 10.1111/cpr.13039 (PMC8168423; doi:10.1111/cpr.13039)
Supplement: Supplementary file 1 — Supplementary Material [file CPR-54-e13039-s001.docx]

Appendix S1

MATERIALS AND METHODS

Cell culture and general materials

We cultured 3T3-L1 cells in Dulbecco’s modified Eagle’s medium (DMEM; GE Healthcare, Chicago, IL, USA) containing 4.5 g/L of D-glucose, 10% fetal bovine serum (FBS), 1% penicillin/streptomycin (P/S), 25 mM 4-(2-hydroxyethyl)-1-piperazineethanesulfonic acid (HEPES), 3.7 g/L of sodium bicarbonate, 4 mM L-glutamine, and 1 mM sodium pyruvate at 37°C in an incubator in a 5% CO_2_ humidified atmosphere.

We purchased rodent diet with 60% kcal of fat from Research Diets (New Brunswick, NJ, USA), AICAR and CC from Selleckchem (Houston, TX, USA), and C29 from Cayman Chemical (Ann Arbor, MI, USA). We also obtained ELISA kits from the following companies: insulin, leptin, adiponectin, and resistin ELISA kits from CUSABIO (Hubei, China) and IFN-γ, interleukin (IL)-6, and monocyte chemoattractant protein-1 (MCP1) from BioLegend (San Diego, CA, USA). In addition, we obtained antibodies from the following companies: AKT, rabbit immunoglobulin G (IgG) isotype, and goat IgG isotype antibodies from Bioss (Woburn, MA, USA); PPARγ, C/EBPα, FABP4, t-AMPKα, p-AMPKα, t-ACC, p-ACC, FAS, p-AKT, t-AKT, t-AS160, p-AS160, and MyD88 antibodies from Cell Signaling Technology (Danvers, MA, USA); adiponectin, Arg1, ATGL, IL-6, TNF-α, and TLR2 antibodies from CUSABIO; SREBP-1c from Novus Biologicals (Centennial, CO, USA); leptin and resistin antibodies from R&D Systems (Minneapolis, MN, USA); and β-actin, glyceraldehyde 3-phosphate dehydrogenase (GAPDH), and SCD1 antibodies from Santa Cruz Biotechnology (Dallas, TX, USA).

L-14 extract preparation

We cultured *L. plantarum* L-14 strain (KTCT13497BP) obtained from NeoRegen Biotech (Gyeonggi-do, South Korea) in MRS agar for 18 h at 37°C for precultivation and then 1%-inoculated it for main cultivation in 500 mL of MRS broth and cultured it for 18 h at 37°C. The cultured L-14 was harvested by centrifuging at 10,000 ×*g* for 10 min at 4°C, washed twice with PBS, and then washed with distilled water to completely remove MRS broth and PBS. Next, L-14 resuspended in 20 mL of distilled water was sonicated on ice for 30 min using a sonicator. To remove cell wall ingredients and other residues, we centrifuged it at 10,000 ×*g* for 20 min at 4°C and discarded the pellet. The supernatant was filtered (0.2 μm), frozen overnight at –80°C, and then freeze-dried and reconstituted with PBS before use. In addition, to identify the properties of the key molecule in L-14 extract, L-14 extract (N60) was adjusted to pH 7.0 (P60) or incubated for 30 min at 90°C (H60).

Differentiation of 3T3-L1 cells and hBM-MSCs

We seeded 3T3-L1 cells or hBM-MSCs in 24-well plates at a density of 1.0 × 10^5^ cells/well in the medium as described earlier. Two days after confluence, the medium was changed with MDI containing alpha–Minimum Essential Medium (MEMα), 10% FBS, 1% P/S, 1 uM dexamethasone, 0.5 mM isobutylmethylxanthine, 100 uM indomethacin, 10 mg/mL of insulin, and L-14 extract, P60, and H60. Four days after the first adipogenic induction, we replaced the medium with adipogenic maintenance medium containing MEMα, 10% FBS, 1% P/S, 10 mg/mL of insulin, and L-14 extract. During adipogenic differentiation, we replaced the medium every 2 days, and 3T3-L1 cells and hBM-MSCs were maintained for 12 and 7 days, respectively. After 12 days, to compare lipid accumulation, 3T3-L1 cells and hBM-MSCs were washed with PBS, fixed with 4% formaldehyde, and stained with Oil red O solution for 30 min. Stained adipocytes were observed under an EVOS CL Core microscope (Life Technologies, Carlsbad, CA, USA) at ×200 magnification. To compare the relative lipid accumulation, the Oil red O stain in 3T3-L1 cells and hBM-MSCs was dissolved in isopropanol and quantified by measuring absorbance at a wavelength of 500 nm using a microplate reader. Results were analyzed as a percentage of controls, considered 1.0. The formula to measure the relative lipid accumulation was (*A*_sample_–*A*_blank_)/(*A*_control_–*A*_blank_). In addition, TAG was quantified using the TAG assay kit (Cayman Chemical) according to the manufacturer’s instructions.

Western blot analysis

We harvested 3T3-L1 cells and hBM-MSCs and lysed them on ice for 5 min in Cell Culture Lysis 1X Reagent (Promega) containing a mixture of protease and phosphatase inhibitors (MCE). Next, the insoluble debris was removed by centrifugation at 15,000 ×*g* for 15 min at 4°C. The total protein concentration was quantified by Pierce^TM^ BCA Protein Assay Kit (Thermo Fisher Scientific, Waltham, MA, USA). We separated 10‒40 μg of proteins in the isolated supernatants using 8‒12% sodium dodecyl sulfate–polyacrylamide gel electrophoresis (SDS-PAGE) and transferred them to polyvinylidene difluoride (PVDF) membranes. The PVDF membranes were incubated in 0.1% Tween 20 tris-buffered saline (TBST) containing 5% bovine serum albumin (BSA) for 1 h at room temperature and then incubated overnight in 5% BSA-TBST with primary antibodies at 4°C. Next, the PVDF membranes were washed thrice with TBST and incubated in 5% BSA-TBST with horseradish peroxidase (HRP)-conjugated secondary antibodies for 1 h at room temperature. Protein signals on the PVDF membranes were developed using ECL western blot analysis substrates and analyzed by GeneGnome XRQ System (Syngene, Cambridge, UK), Fusion FX6.0 (Vilber, Collégien, France), and Medical X-ray film blue (AGFA, Mortsel, Belgium). All experiments were repeated three times.

Animals, diets, and study design

We purchased 4-week-old C57BL/6J male mice from Orient Bio (Seongnam, South Korea) and randomly divided them into three groups: (i) normal diet (ND; *n* = 6), (ii) HFD diet (*n* = 7), and (iii) HFD+L-14 extract (*n* = 8). The mice were fed ND or HFD for additional 7 weeks, with free access to water. In the HFD+L-14 group, L-14 extract (500 mg/kg of body weight) was orally administered using a feeding needle catheter every 2 days, while in ND and HFD groups, PBS was orally administered under the same conditions to induce the same stress. The body weight and food consumption were measured every 2 days. After 7 weeks of feeding and administration, the mice were fasted overnight and then euthanized. Epididymal and iWAT was collected and weighed, and liver and blood serum were immediately separated for further analysis. Total proteins from the WAT and liver were isolated using SuperFastPrep-2™ (MP Biomedicals, Irvine, CA, USA), and western blot analysis was performed, as described before. Finally, biochemical analysis of serum was performed by the Korea Mouse Phenotyping Center (Seoul, South Korea), and hormones and cytokines in serum were quantified using the ELISA kit according to the manufacturer’s instructions.

Histology of mouse eWAT and liver

Mouse eWAT and liver were rinsed twice with sterilized PBS, fixed in 4% PFA in PBS overnight, embedded into paraffin blocks, cut into 5-μm-thick slices, and placed on adhesive microscope slides. The dewaxed and rehydrated sections were stained by H&E. For IHC, antigens were retrieved from the sections in pressure vessels filled with citrate buffer (pH 7.0) for 10 min, and the sections were immersed in BLOXALL® Endogenous Peroxidase Solution (Vector Laboratories, Burlingame, CA, USA) for 20 min at room temperature and incubated in 2.5% normal horse serum to reduce nonspecific binding. Next, primary antibodies were diluted 1:100‒200 with 2.5% normal horse serum, and the sections were again incubated with the diluted antibodies overnight at 4°C. Rabbit and goat IgG antibodies were used for negative controls. Subsequently, the sections were incubated with the ImmPRESS Polymer Anti-Rabbit and Anti-Goat IgG reagents for 30 min at room temperature, stained with ImmPACT® DAB Peroxidase (HRP) Substrate (Vector Laboratories), and then lightly counterstained by hematoxylin. Finally, images were obtained under an Olympus BX50 microscope (Olympus, Tokyo, Japan).

Analysis of the effects of L-14 extract and EPS on AMPK and TLR2 signaling pathways

We seeded 3T3-L1 cells in 24-well plates at a density of 1.0 × 10^5^ cells/well in the medium, as described before. Two days after confluence, we replaced the medium with starvation medium containing DMEM and 1% P/S for 1 h. After starvation, we replaced the medium with normal medium containing 250 μM AICAR, 5 μM CC, and 50 μM C29. Next, the cells were incubated for 2 h and then induced to differentiate into mature adipocytes in MDI with L-14 extract or EPS, as described before. AICAR and C29 treatment was performed every 2 days, while CC was not further used during the entire period. After 12 days, we analyzed the adipogenic inhibitory effect of L-14 extract or EPS via AMPK and TLR2 signaling pathways using Oil red O staining and TAG assay. In addition, to determine the effects of L-14 extract or EPS during the early stage of adipogenic differentiation, we isolated proteins on day 4 and analyzed them by western blot analysis.

EPS purification

We sonicated the cultured L-14, as described before. We added trichloroacetic acid to reach a final concentration of 14% (v/v) to denature the protein. Next, we incubated L-14 extract for 30 min at 37°C in a shaking incubator at 90 rpm and centrifuged it at 8000 ×*g* for 20 min. The supernatant was collected, cold absolute ethanol was added to reach a final concentration of 67% (v/v), the mixture was incubated for 24 h at 4°C, and the precipitate was collected. We added distilled water of the same volume as the precipitate, dialyzed the solution using Standard RC tubing (molecular weight cut-off = 3.5 kDa; Spectrum Chemical, New Brunswick, NJ, USA) for 2 days with water changes twice a day, and filtered (0.2 μm) the dialysate. To identify EPS as a homogeneous polysaccharide, we separated 30 mg/mL of EPS using size-exclusion chromatography on a HiLoad® 16/600 Superdex 200 pg column (GE Healthcare) with PBS, analyzed it using the ÄKTA FPLC system (GE Healthcare), freeze-dried it, and then reconstituted it with PBS before use. To measure the ratio of EPS in L-14 extract, the quantified L-14 extract was resuspended in 10 mL PBS, and EPS was isolated as described above. The ratio of EPS in L-14 extract was represented as (EPS mass/L-14 extract mass) × 100.

Table

Table S1. Primer sequences used for RT-qPCR

|  | **Genes** | **5’ -> 3’** | **Sequence** |
| --- | --- | --- | --- |
| Mouse primers | PPARγ | Forward | TTCAGAAGTGCCTTGCTGTG |
|  |  | Reverse | GCTGGTCGATATCACTGGAGA |
|  | C/EBPα | Forward | GGTGCGTCTAAGATGAGGGA |
|  |  | Reverse | CCCCCTACTCGGTAGGAAAA |
|  | FABP4 | Forward | AAGGTGAAGAGCATCATAACCCT |
|  |  | Reverse | TCACGCCTTTCATAACACATTCC |
|  | LPL | Forward | ATGGATGGACGGTAACGGGAA |
|  |  | Reverse | CCCGATACAACCAGTCTACTACA |
|  | FAS | Forward | ATCCGGAACGAGAACACGATCT |
|  |  | Reverse | AGAGACGTGTCACTCCTGGACTT |
|  | GPDH | Forward | ATGGCTGGCAAGAAAGTCTG |
|  |  | Reverse | CGTGCTGAGTGTTGATGATCT |
|  | CD36 | Forward | AGATGACGTGGCAAAGAACAG |
|  |  | Reverse | CCTTGGCTAGATAACGAACTCTG |
|  | GAPDH | Forward | AGGTCGGTGTGAACGGATTTG |
|  |  | Reverse | TGTAGACCATGTAGTTGAGGTCA |
| Human primers | PPARγ | Forward | ACCAAAGTGCAATCAAAGTGGA |
|  |  | Reverse | ATGAGGGAGTTGGAAGGCTCT |
|  | C/EBPα | Forward | AACACGAAGCACGATCAGTCC |
|  |  | Reverse | CTCATTTTGGCAAGTATCCGA |
|  | FABP4 | Forward | ACTGGGCCAGGAATTTGACG |
|  |  | Reverse | CTCGTGGAAGTGACGCCTT |
|  | Leptin | Forward | TGCCTTCCAGAAACGTGATCC |
|  |  | Reverse | CTCTGTGGAGTAGCCTGAAGC |
|  | GPDH | Forward | CTATACAGCATCCTCCAGCACAA |
|  |  | Reverse | GGCCCTCGTAGCACACCTT |
|  | CD36 | Forward | CTTTGGCTTAATGAGACTGGGAC |
|  |  | Reverse | GCAACAAACATCACCACACCA |
|  | GAPDH | Forward | TGGACTCCACGACGTACTCA |
|  |  | Reverse | ACATGTTCCAATATGATTCC |
